# Diving into the brain: deep-brain imaging techniques in conscious animals

**DOI:** 10.1530/JOE-20-0028

**Published:** 2020-05-07

**Authors:** Pauline Campos, Jamie J Walker, Patrice Mollard

**Affiliations:** 1 College of Engineering, Mathematics and Physical Sciences, University of Exeter, Exeter, UK; 2 EPSRC Centre for Predictive Modelling in Healthcare, University of Exeter, Exeter, UK; 3 Bristol Medical School, Translational Health Sciences, University of Bristol, Bristol, UK; 4 IGF, University of Montpellier, CNRS, INSERM, Montpellier, France

**Keywords:** neuroendocrinology, whole animal physiology, deep-brain imaging, neuronal activity, conscious animals

## Abstract

In most species, survival relies on the hypothalamic control of endocrine axes that regulate critical functions such as reproduction, growth, and metabolism. For decades, the complexity and inaccessibility of the hypothalamic–pituitary axis has prevented researchers from elucidating the relationship between the activity of endocrine hypothalamic neurons and pituitary hormone secretion. Indeed, the study of central control of endocrine function has been largely dominated by ‘traditional’ techniques that consist of studying *in vitro* or *ex vivo* isolated cell types without taking into account the complexity of regulatory mechanisms at the level of the brain, pituitary and periphery. Nowadays, by exploiting modern neuronal transfection and imaging techniques, it is possible to study hypothalamic neuron activity *in situ*, in real time, and in conscious animals. Deep-brain imaging of calcium activity can be performed through gradient-index lenses that are chronically implanted and offer a ‘window into the brain’ to image multiple neurons at single-cell resolution. With this review, we aim to highlight deep-brain imaging techniques that enable the study of neuroendocrine neurons in awake animals whilst maintaining the integrity of regulatory loops between the brain, pituitary and peripheral glands. Furthermore, to assist researchers in setting up these techniques, we discuss the equipment required and include a practical step-by-step guide to performing these deep-brain imaging studies.

## Introduction

For almost a century, neuroendocrinologists have worked towards characterising the central regulation of endocrine axes that ensure critical functions such as reproduction, growth, and metabolism. While research questions have for many years focused firmly on the neural control of the pituitary gland, the neuroendocrinology field has grown wider and now includes studying the effect of centrally produced hormones on various brain areas, as well as the role of several peripherally born peptides that affect neuroendocrine systems controlling metabolism. An unavoidable feature of all neuroendocrine systems is that they generate rhythms and rely on these rhythms to function optimally. These hormone oscillations dynamically regulate gene transcription and synaptic transmission, and changes in these oscillations are observed in a variety of (patho)physiological states (Le Tissier *et al.* 2017).

Although it has long been postulated that neuroendocrine neurons have the ability to generate hormone rhythms via the patterning of their electrophysiological activity, the regulatory mechanisms underlying this rhythmic mode of hormone secretion have remained hazy. One reason for this is that research efforts in this area have simply been hindered by the complexity and inaccessibility of the hypothalamic–pituitary system. Another reason is that the study of neuroendocrine function has been largely dominated by techniques that consist of studying isolated cell types in *in vitro* or *ex vivo* preparations. This insular approach has led to an inevitable bottleneck where data on cellular and biochemical processes within specific cell types or nuclei have multiplied without a clear/tangible link to physiological function. Similarly, recent efforts in single-cell transcriptomics of the hypothalamus (Romanov *et al.* 2017, Wang & Ma 2019) have documented a highly complex heterogenous hypothalamus, but the implication of neuropeptide expression for physiological function remains difficult to interpret without appropriate tools. Nevertheless, technological developments in genetics and systems neuroscience have enabled specific neurons to be manipulated *in vivo*. Chemogenetics and optogenetics (Fenno *et al.* 2011) tools have made it possible to study the hypothalamus in ways that were previously unimaginable, shedding light on gonadotropin pulse generation (Campos & Herbison 2014, Han *et al.* 2015, Voliotis *et al.* 2019) and brain control of appetite for example (Atasoy *et al.* 2012, Betley *et al.* 2015). Likewise, genetically encoded calcium indicators (GECIs) (Pérez Koldenkova & Nagai 2013) are now frequently used to monitor neuronal activity in living animals. Their characteristics making them an excellent proxy for electrical activity and a versatile tool that facilitates diverse imaging techniques. For example, fibre photometry experiments that consist of monitoring the average calcium activity of a neuronal population *in vivo* have shown that arcuate kisspeptin neurons are responsible for the generation of pulses of LH (Clarkson *et al.* 2017).

**Figure 1:**
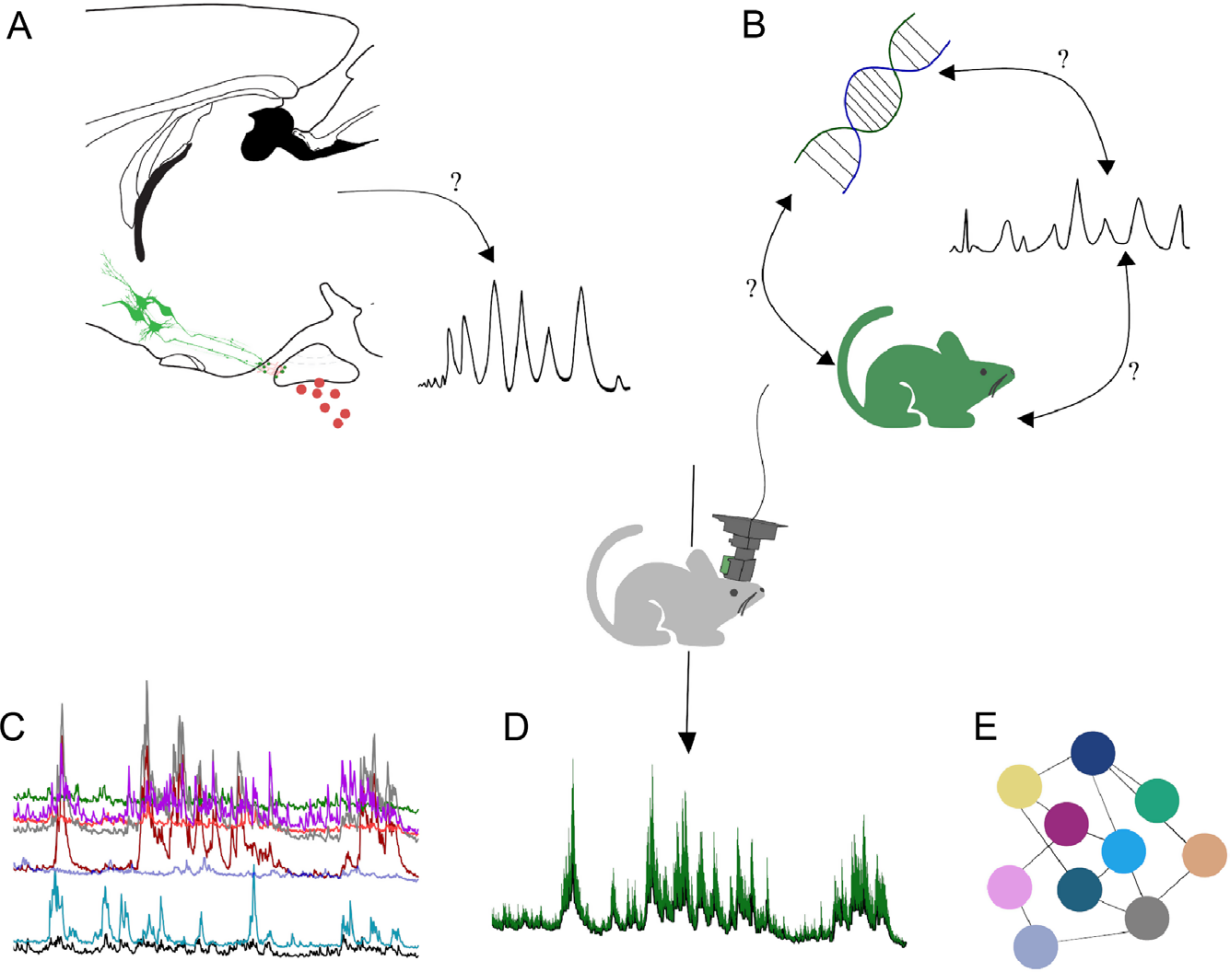
Deep-brain imaging as a powerful tool to understand neuroendocrine functioning. Unknowns such as the relationship between the activity of specific neurons and the dynamics of peripheral hormonal secretion (A) or the way newly discovered genetic mutations result in phenotypic changes (B) can be elucidated using *in vivo* deep brain imaging. Researchers will greatly benefit from real-time visualisation of single neuron calcium activity (C), population calcium activity (D), and will gain insight into the network activity of genetically defined neurons (E).

Despite the valuable insights these techniques have provided, none of them have allowed researchers to study neuronal activity across a population at the single-cell level – characterising for example cell-to-cell heterogeneity or synchronicity – and in turn relate this activity to specific functions. Fortunately, deep-brain single-cell imaging can now be achieved using gradient-index (GRIN) lenses that are chronically implanted and permit imaging of multiple (10–100s) neurons within the population (Barretto *et al.* 2009). Depending on the research question and image quality required, visualisation of neuronal activity can be carried out in head-fixed configuration using a bench-top microscope (Kim *et al*. 2008, Bocarsly *et al.* 2015) or in freely moving configuration using a miniature head-mounted microscope (Ghosh *et al.* 2011). Importantly, using these techniques, it becomes possible to correlate the impact of neuronal activity within a network on other functions. This methodology has been successfully used to study arcuate nucleus and amygdala control of feeding behaviour in freely moving mice (Betley *et al.* 2015, Jennings *et al.* 2015). Similarly, head-fixed microscopy through GRIN lenses has made it possible to image and manipulate pituitary cells over a period of days to weeks in awake mice (Hoa *et al.* 2019) and while it is yet to be published, one can easily imagine combining this technique with serial blood sampling to understand the link between the activity of specific hypothalamic neurons and the resulting peripheral hormonal release (Fig. 1). In the near future, *in vivo* deep-brain imaging could become critical when combined with other emerging strategies. For example, researchers now have the ability to visualise *in vivo* the effect on neuronal activity of individual genomic variants identified from human genomes, thus filling a gap in our general view of neuroendocrine systems (Fig. 1).

**Figure 2:**
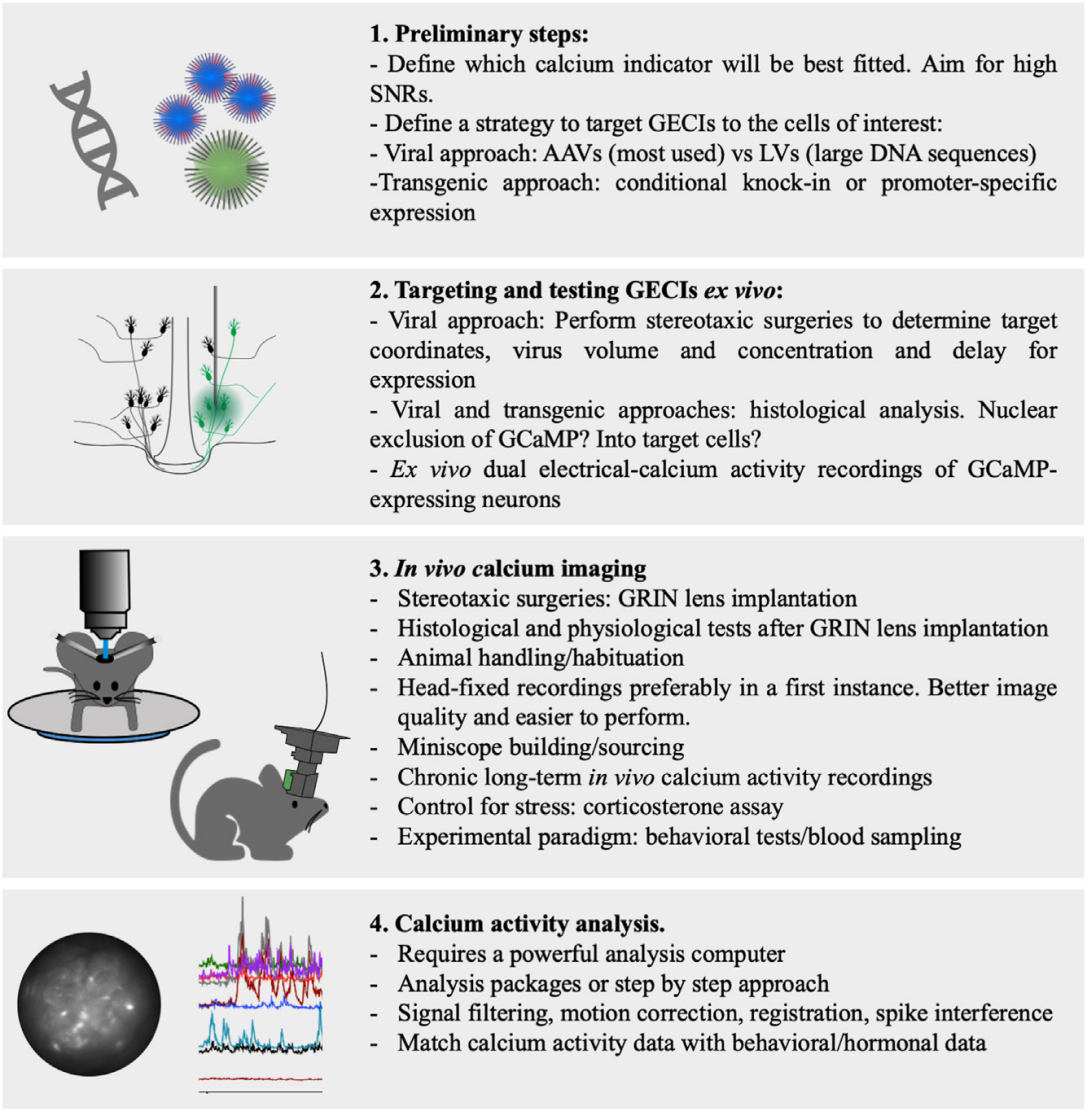
Typical step-by-step approach toward *in vivo* deep-brain calcium imaging.

In this review, we will discuss deep-brain imaging techniques that enable the study of endogenous brain activity from neuroendocrine populations in awake animals whilst maintaining the integrity of regulatory loops between the brain, pituitary and peripheral glands. Rather than cataloguing all available methodologies, we will focus on the most widely used and accessible systems. We will present a step by step approach toward *in vivo* deep-brain imaging (Fig. 2) and provide advice on neuronal activity indicators, neuronal targeting, microscopy techniques, analysis, animal handling and physiological/behavioural assessments so that researchers will be able to set up these techniques to address their own research questions.

## Monitoring neuronal activity with genetically encoded indicators

In order to understand brain processing, scientists have tried to decrypt the action potential (AP) firing of groups of neurons and relate this to specific physiological functions. Traditional methods such as intracellular patch-clamp limit the number of neurons that can be studied at the same time, are invasive and difficult to perform *in vivo*. Extracellular recordings are easier to make, but do not give precise information about the electrical activity of individual neurons and do not enable the study of specific neuronal firing within a population. Looking to overcome these issues, Francis Crick proposed in 1999 that ‘One way-out suggestion is to engineer these neurons so that when one of them fires it would emit a flash of light of a particular wave length’ (Crick 1999); and indeed, the development of genetically encoded optical indicators of neuronal activity has enabled progress towards this goal to an extent that was unimaginable two decades ago. Nowadays, optical imaging enables thousands of neurons to be simultaneously observed *in vitro* or *in vivo*, whilst voltage and calcium indicators reveal spatiotemporal activity patterns within neurons such as dendritic integration, voltage propagation, or dendritic spiking (Lin & Schnitzer 2016). Although microscopy requires optical access to the tissue of interest, many techniques allow minimally invasive optical access or placement of optical probes up to millimetres away from the imaged cells, thus allowing the immediate vicinity of cells under study to be left unperturbed (Ghosh *et al.* 2011, Betley *et al.* 2015).

### Calcium indicators

Calcium is a universal second messenger, playing an essential role in excitable cells and signal transduction. In neurons, APs trigger large and rapid calcium (Ca^2+^) influx through voltage-gated channels. AP firing can therefore be assessed by measuring changes in concentrations of intracellular Ca^2+^ (Yasuda *et al.* 2004). Similarly, activation of neurotransmitter receptors during synaptic transmission causes Ca^2+^ transients in dendritic spines, and with appropriate indicators, imaging intracellular Ca^2+^ dynamics can be used to measure neuronal spiking and synaptic activity across populations of neurons *in vitro* and *in vivo*. GECIs and small-molecule calcium-sensitive dyes (Cobbold & Rink 1987) such as fura-2 (Grynkiewicz *et al.* 1985) are both used to report changes in intracellular Ca^2+^ concentration; but GECIs have the advantage that they enable long-term, repeated non-invasive imaging of specific cells and compartments (Mao *et al.* 2008). State-of-the-art GECIs include Förster Resonance Energy Transfer (FRET)-based sensors (Fiala *et al.* 2002, Palmer & Tsien 2006, Mank *et al.* 2008), but for *in vivo* experiments particularly, the single-wavelength sensor GCaMP family has become the default tool (Tian *et al.* 2009). GCaMPs are based on circularly permuted green fluorescent protein (cpGFP), calmodulin (CaM), and the Ca^2+^/CaM-binding ‘M13’ peptide. Ca^2+^ binding to the calmodulin domain results in a structural shift that enhances the fluorescence by deprotonation of the fluorophore. Since the original GCaMP sensor was published (Nakai *et al.* 2001), many variants have been developed with the purpose of performing calcium imaging *in vivo* (Ohkura *et al.* 2005, Tallini *et al.* 2006, Tian *et al.* 2009). The experimenter will be burdened with picking the appropriate GECI from a broad assortment, but generally speaking, imaging from large populations of neurons in densely labelled samples is helped by lower baseline fluorescence indicators, which reduces the background signal from neuropil and inactive neurons (Dana *et al.* 2019). Moreover, low baseline fluorescence becomes especially important when working *in vivo* where large tissue volumes contribute to the background signal. For the reasons mentioned above, we and others have worked with GCaMP6s,m,f (Chen *et al.* 2013) and are now experimenting with JGCaMP7f (Dana *et al.* 2019); all offer high signal-to-noise ratio (SNR) and GCaMP6m and JGCaMP7 show improved detection of individual spikes (Chen *et al.* 2013, Dana *et al.* 2019). It is also worth noting the development of red-shifted GECIs (e.g. jRCaMP1a, jRCaMP1b, jRGECO1a, jRGECO1b) (Akerboom *et al.* 2013, Dana *et al.* 2016, Montagni *et al.* 2019); these allow the simultaneous use of optogenetic activation of channel rhodopsins using blue light while recording calcium activity (Akerboom *et al.* 2013).

### Voltage indicators

Voltage imaging can directly display membrane potential dynamics and has been used to measure neural dynamics for decades (Bando *et al.* 2019). Genetically encoded voltage indicators (GEVIs) can be categorised into three classes based on their molecular structure and voltage-sensing mechanism: voltage-sensitive domain-based sensors, rhodopsin-based sensors, and rhodopsin-FRET sensors (see Bando *et al.* (2019) for a comparative review on GEVIs). While imaging voltage using fluorescent-based sensors appears to be an ideal technique to probe neural circuits with high spatiotemporal resolution, due to insufficient SNR, imaging membrane potential is still challenging, and in order to achieve single action potential resolution *in vivo*, brighter voltage indicators will need to be developed (Aharoni & Hoogland 2019).

## Directing GECIs to neurons of interest

### Viral vectors

Viruses can be used to target Ca^2+^ indicators to specific cells *in vivo*. Adeno-associated virus (AAV) and lentivirus (LV) are the most common viral vectors used today for neuroscience applications and result in robust expression of the desired gene throughout the cell. Due to their low immunogenicity, AAVs and LVs are well tolerated and highly expressed over long periods of time without any reported adverse effects (Kaplitt *et al.* 1994, Sakuma *et al.* 2012, Samulski & Muzyczka 2014) in various species (Vasileva & Jessberger 2005). AAVs are the most widely used by the scientific community; their efficiency has been evaluated for many brain regions and cell types, and most of them are commercially available from facilities such as Addgene, Penn Vector Core, or ETH Zurich. It is very common to deliver a Cre-dependent viral vector in a Cre-line mutant mouse. With this approach, Ca^2+^ indicator cell-type specificity is typically determined by the genetically controlled presence of a recombinase (Cre) in the specific cells of interest. The viral approach has several advantages, including topographic specificity and the ability to control the amount of protein transduced (adjustment of volume and concentration of viral vectors). However, viral neuronal transfection relies on stereotaxic injection and therefore every animal is unique. Viral vectors also have to be carefully selected and require rigorous* post hoc* histological validation.

#### AAVs

AAVs are small particles (20 nm) allowing them to spread very efficiently within tissues and are particularly well-adapted to brain cell transfection. The choice of the appropriate AAV needs be based on multiple factors, from the physical location of the viral injection in the brain to the serotype/cell-type affinity of the chosen viral vector, as well as the molecular and genetic machinery driving its replication/integration. AAVs present as different serotypes meaning that their capsid proteins differ as well as DNA sequences (inverted terminal repeats, ITRs) that result in variations in speed of viral gene expression onset and target cell specificity. Overall, AAV1, 2, 5, 6, 8, and 9 have different affinities for different cells but should all be able to transfect neurons (Aschauer *et al.* 2013, Hammond *et al.* 2017). When in doubt, AAV-DJ (Grimm *et al.* 2008) or AAV DJ8 (Hashimoto *et al.* 2017) may be good options to test out transfection of neurons that have not been studied before due to their alleged increased tissue tropism. Several ubiquitous promoters are available to drive transgene expressions with AAVs; although not exhaustive, Table 1 summarizes serotypes and promoters used to successfully transfect various hypothalamic neuronal populations.

**Table 1:** Virus database for the transfection of hypothalamic neurons.

Serotype	Promoter	Transgene	Host Specie	Brain area	Neuronal Identity	References
9	EF1a	ChR2	mouse, rat	MS, rPOA, AHA	GnRH	(Campos & Herbison 2014) and unpublished data
9, 5, 2	EF1a, CAG	ChR2, GCaMP6s	mouse	ARN	GHRH	Unpublished data
9, 5, 2	EF1a, CAG	ChR2, GCaMP6s	mouse, rat	PVN, LH	CRH	(Pomrenze *et al.* 2015, Füzesi *et al.* 2016, Romanov *et al.* 2017)
9, 8, 5, 2	EF1a, CAG, Syn	ChR2, GCaMP6s GCaMP6m, hM3Dq	mouse	PVN,	TRH	(Krashes *et al.* 2014) and unpublished data
9	EF1a	ChR2, GCaMP6s, ArchR, HaloR hM3Dq, hM4Di	mouse	RP3V, ARN	Kisspeptin	(Han *et al.* 2015, Clarkson *et al.* 2017)
5	CAG	hM4Di	mouse	ARN	POMC	(Atasoy *et al.* 2012)
1, 8	Syn	ChR2, ArchR, GCamp6s, hM4Di	mouse	ARN	AgRP	(Atasoy *et al.* 2012, Krashes *et al.* 2014, Betley *et al.* 2015)
1, DJ,9, 5	Syn, Ef1a,	GCamp6s, ChETA, hM3Dq, eYFP	mouse	LH, ARN	GABAergic	(Jennings *et al.* 2015, Resendez *et al.* 2016, Silva et al. 2019)
1	Syn	GCamp6s	mouse	VMH	Galanergic	(Viskaitis *et al.* 2017)
1	Syn	GCamp6s	mouse	VMH	Estrogen receptor 1	(Remedios *et al.* 2017)

*Brain area abbreviations:* AHA, anterior hypothalamus; ARN, arcuate nucleus; LH, lateral hypothalamus; MS, medial septum; PVN, paraventricular nucleus; RP3V, rostral periventricular region of the third ventricle; rPOA, rostral preoptic area; VMH, ventro-medial hypothalamus.

*Neuronal identity abbreviations:* AgRP, Agouti-related peptide; CRH, corticotropin-releasing hormone; GABA: gamma-aminobutyric acid; GHRH, growth hormone-releasing hormone; GnRH, gonadotropin-releasing hormone; POMC, pro-opiomelanocortin; TRH, thyrotropin-releasing hormone.

#### Lentiviruses

LVs are big particles (80–120 nm) and can carry inserts as large as 10 kb (as opposed to the limited 4.5 kb capacity of AAVs), which is particularly pertinent when working with a promoter-specific virus. Although they are less commonly used than AAVs, LVs are more efficient at transfecting cells *in vitro*, have similar capabilities *in vivo* (but do not spread as widely as AAVs) and do not require making a choice of serotype (Cannon *et al.* 2011).

### Transgenics

In order to work with animals with similar levels of transgene expression within the targeted neuronal phenotype, transgenic lines have been generated. Essentially, two approaches exist.

In the first type of transgenic line, GCaMP is expressed under the control of specific promoters. For example, the *Thy1-GCaMP3* (Chen *et al.* 2012), *6s* and *6f* (Dana *et al.* 2014) transgenic mouse lines express GCaMP in Thy1-expressing neurons across the entire brain and have been used for *in vitro* and *in vivo* Ca^2+^ imaging. Similarly GCaMP2 expression under the Kv3.1 potassium-channel promoter has been used to perform Ca^2+^ imaging of the glomerular layer of the mouse olfactory bulb (Fletcher *et al.* 2009). Finally, transgenic rat lines have been developed and *Thy1-GCaMP6f* rats have been used to record cortical activity with a head-mounted microscope (Scott *et al.* 2018).

The second and more common approach is to generate transgenic animals bearing GCaMP expression in specific cell types, by crossing a Cre-driver with a floxed-GCaMP mouse (Hsiang *et al.* 2017, Zimmerman *et al.* 2019). Several floxed-GCaMP mice lines are now commercially available from Jackson Laboratories and have been characterised by users. Nevertheless, potential concerns have to be kept in mind if working with transgenics and attention will need to be given to potential ectopic expression, drift and loss of specificity of the transgene, or even disrupted Ca^2+^ dynamics within GCaMP-expressing neurons (Steinmetz *et al.* 2017). After years of working with GECIs or genetically-addressable opsins, many labs including our own have favoured the use of viruses. Overall, viral vectors lead to a greater level of expression of the desired transgene, which is essential *in vivo*, and generating transgenic lines has been an expensive, time-consuming process that has not always resulted in success.

### Histological validation of GCaMP expression

Regardless of the technique used to target GCaMP to specific cells, it is essential to confirm the extent of transgene expression, spread, and location over the experimental timeline using histology methodologies. Typically, animals will be terminally anesthetized and transcardially perfused with a 4% paraformaldehyde solution, and brains will be removed, sectioned and processed for histological analysis. Native GCaMP expression should be bright enough to visualize in fixed sections with epifluorescence or confocal microscopy, with fluorescence signal limited to the cell membrane. Filled nuclei and/or misshapen neurons can be a sign of overexpression, which can be overcome by reducing viral vector concentration.

In order to identify transfected neurons with immunohistochemistry techniques, GCaMP labelling can be achieved using a GFP antibody; this is especially useful in situations where GCaMP cannot be clearly detected without enhancement. Alternatively, it is possible to perfuse the animal with calcium-containing DPBS (Dulbecco’s phosphate buffer saline). Since DPBS contains a saturating concentration of Ca^2+^ (0.9 mM), GCaMP brightness will be maximal, and the experimenter may therefore be able to visualize GCaMP fluorescence without antibodies (Dana *et al.* 2014).

## Imaging deep brain areas

While cortical activity can be recorded through cranial windows, hypothalamic neuroendocrine neurons are located deep in the brain (from half a centimetre deep for mice to up to a centimetre deep for rats) and it is therefore necessary to find tools that allow the experimenter to see ‘deep inside’ the brain. Fortunately, in parallel to the development of Ca^2+^ indicators, instrumentation has been optimized to render Ca^2+^ imaging possible in subcortical brain areas.

### GRIN lenses

GRIN lenses resemble simple glass needles, but they are in fact complex lenses with a radially varying index of refraction; this causes an optical ray to follow a sinusoidal propagation path through the lens. GRIN lenses combine refraction at the end surfaces along with continuous refraction within the lens. Their characteristics allow them to relay optical images from one end to the other, with very low aberrations for objects near the optical axis. For these reasons, GRIN lenses are the perfect tool to perform *in vivo* imaging in deep-brain regions, relaying signals to a point above the skull surface where image acquisition can take place (Barretto *et al.* 2009). GRIN lenses exist in different lengths and diameters. For brain regions such as the dorsal striatum (Zhang *et al.* 2019), prelimbic cortex (Pinto & Dan 2015) or hippocampus (Ziv *et al.* 2013), a 1 mm diameter GRIN lens can be used to increase the field of view. For deep-brain areas such as the amygdala (Li *et al.* 2017) or hypothalamus (Jennings *et al.* 2015), a lens of approximatively 0.5 mm diameter can be used in order to minimize tissue damage during lens implantation. For neuroendocrine hypothalamic areas (e.g. arcuate nucleus, paraventricular nucleus, rostral preoptic area), we have successfully used 0.6 mm diameter 7.4 mm long lenses for mice, and 0.8 mm diameter 10.8 mm long lenses for rats.

### Prism probes

Side-view GRIN lenses or ‘prism-probes’ exist. These are composed of a prism fixed to the bottom of a cylindrical GRIN relay lens. The slanted surface of the prism is coated with metal (aluminium or silver for high reflectivity) to reflect by 90° the light from the microscope to excite GCaMP cells located along the imaging face of the prism probe. The emitted light from the cells also reflects off the hypotenuse of the prism and can be collected through the objective of the microscope. These probes have been used for multi-layer cortical imaging (Gulati *et al.* 2017) and could be suitable for populations of hypothalamic neurons that are spatially distributed along the z-axis.

### GRIN lens implantation

Unravelling the intricacies of neuroendocrine function with *in vivo* imaging studies lasting days to weeks requires optical access to the structure of interest while maintaining both its integrity and that of the surrounding tissue. First of all, before performing GRIN lens implantation in animals, it is sensible to use a ‘phantom brain’ preparation where the GRIN lens is implanted into an agar/fluorescent microbead preparation (Kim *et al.* 2017). This preparation can be looked at under any epifluorescence microscope in order to verify the working distance of the GRIN lens. GRIN lenses have to be placed above the targeted structure and the space needed between the lens and the neurons is determined by the working distance (50–350 μm) of the imaging end of the GRIN.

In order to gain optical access to the hypothalamus, a GRIN lens has to be guided through the cortex towards the ventral side of the brain. To overcome the challenge of crossing the meninges and going through the brain tissue without increasing pressure and causing excessive damage, the GRIN lens can be inserted into the lumen of a needle, which can then be retracted once the GRIN lens is located correctly. Practically, the GRIN lens is placed inside a small gauge needle, with movement along the needle restricted by placing a metal rod above it. The needle is then lowered to a point just above the target, and then removed with the metal rod kept in place, ensuring the GRIN lens stays in place. Finally, the metal rod is removed and the GRIN lens fixed in position with dental cement (Hoa *et al.* 2019). An alternative is to first create a path to the target structure with a needle or a dissection knife (Gulati *et al.* 2017), and to then lower the naked GRIN lens along this path; in this method, the lens can be carefully held using a bulldog clamp or a pipette tip trimmed to match the diameter of the lens. Another interesting method consists of placing the top part of the GRIN lens inside a glass tube and fixing it using Super Glue. The glass tube can then be held with an electrode holder on the stereotaxic arm. Once the GRIN lens has been positioned in the brain and cemented in place, acetone can be carefully introduced into the glass tube in order to dissolve the Super Glue, thus freeing the GRIN lens (Zhang *et al.* 2019).

GRIN lens implantations are invasive procedures that cause inflammatory responses in the brain, during which the neurons do not behave normally. It is recommended to wait at least 3 weeks after implantation before starting imaging sessions; overall, we have had the most success imaging Ca^2+^ activity from 5 weeks to several months after GRIN lens implantation. It is also necessary to ensure that GRIN lens implantations do not result in long-term inflammatory responses that could alter physiological function. Attention therefore needs to be given to possible behavioural/hormonal changes, and* post hoc* histological validation is necessary.

### Sourcing GRIN lens

Currently, to our knowledge, there are two manufacturers of GRIN lenses suited to brain imaging applications. The first is GRINTECH, who hold a strategic partnership with the neurotechnology company Inscopix. All GRINTECH lenses used in the field of non-confocal, single photon epi-fluorescence imaging for non-human neuroscience application must be purchased through Inscopix. The second manufacturer is GoFoton, who are the largest manufacturer of GRIN lenses in the world. Although their GRIN lens production has been focused on telecommunication applications and fibre coupling, they have been working over the past year at developing GRIN lenses specifically for brain imaging applications. Hopefully, GoFoton will be able to provide high-quality GRIN lenses for microscopic applications in the near future.

## Imaging in conscious animals

Deep-brain imaging of Ca^2+^ activity can be performed through GRIN lenses that are chronically implanted; these relay fluorescence captured inside the brain to the surface of the skull, which can be observed with conventional microscopy techniques. Depending on the research question and the image quality needed, deep-brain imaging can be performed with a bench top microscope on head-restrained animals or with the help of a head-mounted miniature microscope for freely-moving animals.

### Head-fixed configuration

**Figure 3:**
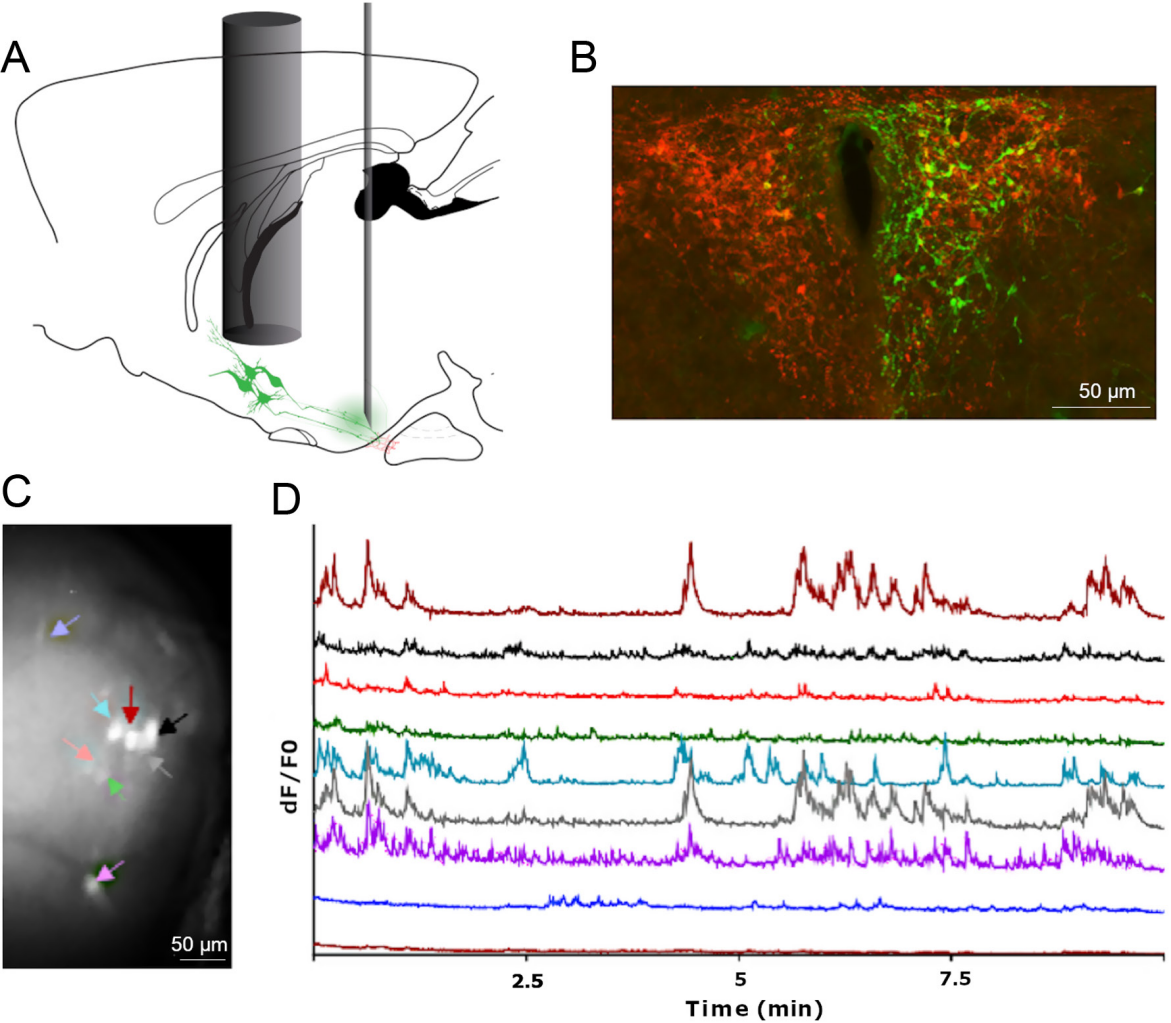
*In vivo* calcium imaging of TRH neurons in conscious mice. (A) *Trh-IRES-Cre* mice were injected in the median eminence with a viral vector (AAV5.CAG.Flex.GCaMP6s.WPRE.SV40) and TRH neurons were visualised through a GRIN lens placed above the PVN. (B) Immunofluorescence image showing GCaMP6s expression (green) in TRH neurons (red) located in the PVN. (C) GRIN lens view of a field of the TRH neurons expressing GCAMP6m. Regions of interest corresponding to individual neurons are indicated by coloured arrows. (D) Time-lapse recordings of calcium (GCAMP6s) activity of eight hypophysiotropic TRH neurons in an adult *Trh-IRES-Cre* male mouse. Scale bars 50 μm.

In order to perform deep-brain imaging in conscious animals, rodents can be temporarily restrained under a microscope with the help of a metal piece chronically fixed to their skull. Animals retain the ability to move on a wheel or a cylinder in order to give an impression of movement and reduce stress during the experiments. This approach has been used to perform calcium imaging in deep-brain areas (Bocarsly *et al.* 2015), and even in two areas at the same time (Lecoq *et al.* 2014). While most of these studies have been performed using two-photon microscopes, we have successfully performed 1-photon calcium imaging in the hypothalamus of head-fixed mice with a stereomicroscope fitted with a 20x objective (for detailed methodology see Hoa *et al.* (2019)). Using this set-up, we investigated the endogenous activity of hypophysiotropic TRH neurons across several days. To do so, we worked with *T* *rh-IRES-Cre* mice (Krashes *et al.* 2014) (kindly gifted by Prof. B Lowell, Beth Israel Deaconess Medical Center & Harvard Medical School) that had been injected with a Cre-dependent AAV driving GCaMP6s expression. We recorded the activity of several TRH neurons at single-cell resolution level in head-fixed mice (Fig. 3). This experimental design enabled us to study how hypophysiotropic TRH neurons interact with each other within local networks and modulate their activity throughout the day (P Campos and P Mollard, unpublished observations). A great benefit of deep-brain imaging in head-fixed configuration is that bench top microscopes offer excellent imaging quality, and it is therefore possible to record the activity of sub-cellular structures (Meng *et al.* 2019). Indeed, we have successfully imaged the calcium activity of assemblies of TRH neuron terminals in the median eminence, although 1-photon microscopy could not spatially resolve individual boutons (P Campos and P Mollard, unpublished observations).

### Miniscopes

In 2011, Mark Schnitzer’s group first reported the miniature microscope (miniscope) with epifluorescent light source (Ghosh *et al.* 2011), which was small, light, and easy to mount on the head of a mouse for studying complex behaviours in freely moving animals. Combined with a GRIN lens, the miniscope imaging system enables recording of calcium activity from hundreds of neurons in deep-brain regions for months, with imaging quality comparable to benchtop microscopes. Over the last 5–10 years, several miniscope designs have emerged, but currently, two miniscopes in particular appear to be widely used: the Inscopix nVista miniscope and the UCLA miniscope. These two miniature microscopes share the same core features. The optical design includes an achromatic lens which focuses near-collimated light from an objective GRIN lens onto a CMOS sensor and a set of optical filters. The plastic housing can be machined or 3D printed, and custom electronics are used to control and read-out the excitation LED light source and CMOS imaging sensor. Miniscopes are connected to a data acquisition (DAQ) box that relays data to a computer. A base plate is attached to the skull of the animal with dental cement and allows magnetic latching of the miniscope to the baseplate.

#### nVista Inscopix miniscope

The Inscopix company was founded in 2011 by Kunal Ghosh, Mark Schnitzer and Abbas El Gamal in order to commercialise their miniature microscope. Some years later, their technology has evolved and the Inscopix miniature microscope, called nVista, is now widely used. The characteristics of the current version of the nVista microscope are presented in Table 2. It possesses a software-controlled electronic focusing mechanism (300 μm) that does not require any manual intervention. The miniscope is connected to a DAQ box which has the capacity to store data on an SSD and/or an SD card. The DAQ box is also equipped with ethernet and WIFI connectivity, which allows remote control of the miniscope and data streaming through Web-based acquisition software.

**Table 2:** Comparison of the characteristics of the Inscopix nVista miniature microscope and UCLA miniscope.

System	UCLA miniscope-3rd generation	nVista 4th generation
Weight (g)	3	1.8
Size L × W × H (mm)	16.3 × 13 × 22.5	8.8 × 15 × 22
Footprint on scull (Baseplate) (mm^2^)	75	57.44
Resolution (pixels)	752 × 480	1280 × 800
Field of view (µm)	700 × 450^a^	650 × 900
Sample-rate (Hz)	60	50
Focusing mechanism	Manual (300 μm) - Slider	Electronic (300 μm) - Liquid lens

^a^The field of view given for the 3rd generation UCLA miniscope is based on imaging superficial brain structures. The addition of an objective GRIN lens that is required to image deep-brain structures reduces this field of view. To our knowledge, precise field of view measurements for deep brain imaging using this miniscope are not known.

On purchasing the nVista system, researchers will receive a full support package, which makes it probably the easiest way to start working with deep-brain imaging with a miniscope. Researchers will benefit from field scientific consultants that will, through site visits and online communication, help them set up the technology and troubleshoot experimental and conceptual challenges (from Ca^2+^ indicator selection, to data analysis) in order to ensure the success of the research project. It is worth highlighting that the nVista miniscope comes with a 1-year manufacturer’s warranty that can be prolonged to 5 years. For damaged scopes sent back to the company, turn-around time is typically around 1 week. In our own experience, this warranty has been valuable; we have had to return damaged nVista miniscopes to Inscopix 3–4 times per year for repair. This support package comes at a price of course; currently, the nVista system (consisting of one nVista 3.0 scope + accessories + 1-year warranty + help + software) costs in the region of 70–80,000 USD.

#### UCLA miniscope

The UCLA miniscope is also based on the design pioneered by Mark Schnitzer’s Lab, but this miniscope is open-source. The characteristics of the UCLA miniscope version 3 (V3) are presented in Table 2. It also uses a single, flexible coaxial cable to carry power, communication, and data transmission to a custom open source DAQ box and software. The default version of the UCLA miniscope only allows cortical imaging. For imaging deeper brain areas, an objective GRIN lens can be mounted inside the miniscope and positioned above a GRIN relay lens chronically implanted above the target cells. Focusing adjustment (300 μm) occurs through a linear slider that can be manually adjusted in height; although it preserves the orientation of neurons between imaging sessions, it can be tricky to use, especially when the miniscope is head mounted.

The UCLA miniscope has had a significant impact on the neuroscience community, with at least 400 labs around the world building and using these imaging devices for their research studies over the past 3 years. This success is likely due to a broad dissemination policy. The ‘miniscope project’ began as a collaboration between the Golshani Lab, Silva Lab, and Khakh Lab at the University of California, Los Angeles, and provides documentation, tutorials on part procurement, assembly and experimental application guides, both online and through workshops. The miniscope website (http://miniscope.org) provides a centralized hub for users to share design files, source code, and other relevant information related to this imaging technique.

Building a UCLA miniscope is cost-effective (DAQ + 1 scope < $1000 USD). It is not complicated but does require soldering and hands-on assembly. A main advantage is that it can be tuned to fit the research project requirements, and additionally by building it, researchers understand how to fix it quickly. For those that do not feel confident building the miniscope, LabMaker (www.labmaker.org), based in Berlin, Germany, sell the UCLA miniscope as a kit that does not require any soldering for approximately $2000 USD (includes DAQ + 1 scope).

#### Improvements of the UCLA miniscope

It is worth noting that this is a rapidly evolving field, with technological improvements in miniature microscopes emerging all the time. For example, at the time of writing, a new version (V4) of the UCLA miniscope has just been released (http://miniscope.org). Compared to the UCLA V3 miniscope, the V4 miniscope is lighter (2.6 g), utilises a smaller baseplate (25 μm footprint), has a field of view that can be set based on the combination of lenses used when assembling the miniscope (1000 μm-diameter or more), has an improved CMOS that requires just a fifth of the excitation power of previous systems, and is expected to have improved resolution (0.5 MP) and faster imaging speed (120FPS) (Daniel Aharoni, 2019 Neuro Open-source Workshop, January 2019). In addition, the manual focusing mechanism of V3 has been replaced by an electronic system. Indeed, similar to the nVista system, the V4 miniscope benefits from liquid lenses that consist of an interface of two immiscible liquids which, under voltage, deform and act as a lens allowing for electronic focusing without the need for manual adjustment. This should make focusing much easier, and because liquid lenses can be adjusted rapidly, it may become possible to perform interleaved recording from different focal planes (Aharoni & Hoogland 2019). Finally, in order to allow for simultaneous two-colour fluorescence imaging, the objective GRIN lenses present in the previous version of the UCLA miniscopes that created chromatic aberrations, have been replaced in V4 miniscopes by achromatic lenses (Aharoni & Hoogland 2019). However, since deep-brain imaging necessitates the implantation of GRIN lenses in the brain, this new feature does not yet represent an advantage for the study of deeply located neuroendocrine cells. It is important to note that although the UCLA V4 miniscope possesses promising improvements and can be fitted with relay GRIN lenses for deep brain imaging, to our knowledge, they have not yet been tested for structures deeper than the cortex. The V4 is expected to be released as a kit in 2020.

### Alternatives and next-generation miniscopes

#### Wire-free scopes

Whilst tethered systems are currently the most effective method for *in vivo* recording/control of freely moving animals, wireless (Liberti *et al.* 2017) and wire-free (Shuman *et al.* 2020) open-source miniscopes have been developed with the latest being used to record CA1 place cells during maze navigation in epileptic mice. These recent studies have demonstrated the feasibility of using a lightweight wireless miniature microscope system to transmit Ca^2+^ imaging data to a receiver (Liberti *et al.* 2017) or save data directly to a microSD card positioned on the miniscope (Shuman *et al.* 2020). Both designs are powered by onboard lithium polymer batteries that can be attached to the microscope body or mounted on the back of the animal, consequently adding weight to the microscope design (~2 g for a 50 mAh battery). Alternatively, wireless powering of a miniaturized design with low power consumption is thought to be feasible with near-field wireless power transfer similar to those that have already been implemented for wireless optogenetic stimulation (Aharoni & Hoogland 2019). While power consumption (which scales with data bandwidth) is one of the limiting factors in designing a miniaturized wireless microscope due to battery size and weight requirements, power-efficient CMOS imaging sensors can be used along with pixel binning or pixel subsampling to minimize power consumption while maintaining a comparable field of view, albeit at a lower resolution than is possible using tethered systems (Aharoni & Hoogland 2019). The open-source nature of ongoing miniscope projects allows for the rapid sharing of designs, modifications and ideas across laboratories, and it is expected that wireless systems will rapidly improve and that their use will soon escalate within the neuroscience community. Of particular interest here is Open-ePhys, a non-profit initiative encompassing a large team of scientists and developers, which has released a kit to convert the wired UCLA miniscope (version 3) into a wire-free system.

#### Two-photon imaging

Two-photon (2P) excitation improves optical sectioning of samples and thereby ensures that the light collected is only from the cellular and subcellular structures that lie along the focal plane of 2P excitation. While 2P brain imaging in animal models usually requires that the animal is head fixed, there have been several successful attempts at building a 2P miniscope (see Helmchen *et al.* 2013 for review) with some designs used to image in freely moving rodents (Zong *et al.* 2017, Ozbay *et al.* 2018). A major disadvantage to this technique is that it requires expensive table-top lasers coupled to optical fibres to achieve 2P excitation (Aharoni & Hoogland 2019). Furthermore, a recent study compared data acquired with a 2P benchtop microscope with one-photon images acquired with a miniscope and found that orientation tuning of the same identified neurons was comparable irrespective of the type of fluorescence excitation used (Glas *et al.* 2019).

## Animal handling

Regardless of the imaging technique, it is important to take the time to handle animals to ensure they become familiar with the experimenter and being moved in and out of their cage. It is possible to quickly and briefly anaesthetise the animal when attaching the miniscope or placing the animal in the head-attached set-up, but it is not recommended. Indeed, as discussed later in this review, anaesthetics, even when used for a very short period of time, may affect neuronal dynamics, lead to changes in hormone secretion, or alter animal behaviour.

### Head-fixed habituation

For head-fixed experiments, it is useful to place the animal under the microscope for a few minutes every day leading up to the experiment. Attention should be paid to details such as lighting, electrical equipment, and ventilation in order for the animal to acclimatise to the exact conditions that will be used for the experiment. It is also important to protect the animal’s eyes from the microscope’s excitation light by covering the front part of the head with suitable opaque material. We have found that animals never get completely used to being head restrained for long periods of time, but studies have reported self-initiated head-fixation system for functional imaging in mice. For example, mice have been shown to self-initiate head-fixation in order to get water, enabling imaging to be performed through cranial windows (Murphy *et al.* 2016, Aoki *et al.* 2017). Although it has never been used for deep-brain imaging, this habituation technique seems promising and shows that with patience and good experimental design, it is indeed feasible to perform imaging in ‘low stress’ head-fixed animals.

### Miniscope habituation

For miniscope experiments, the acclimatisation process is more straightforward. Animals should first habituate to their imaging arena for a few minutes each day leading up to the experiment. Then, it is important for the animals to get used to carrying the miniscope, which can weigh about 10% of the weight of an adult mouse. We use ‘dummy’ miniscopes to train our animals; they are of the exact same weight and size as the real miniscope and are connected using the same type of cable. We have found that animals carrying dummies for a few days soon behave in a completely normal way. It is also useful to use a dummy miniscope so the animals get used to the microscope cable. Indeed, mice will try to chew on the cable, and we have found that coating the dummy cable with bitter nail polish usually prevents mice from chewing the real cable during experiments. We have yet to try this methodology with rats.

### Evaluating stress

Together with handling and habituation, it is recommended to evaluate the level of stress of the animals throughout the experiments by measuring corticosterone concentration. Commercially-available ELISA kits are widely used to quantify corticosterone levels for the assessment of stress in laboratory animals. Some of them, such as the AssayMax corticosterone ELISA kit (AssayPro), only require small volumes of whole blood (3–6 μL per sample) that can be collected using a minimally invasive technique such as tail-tip blood sampling (Guillou *et al.* 2015). Although the precision in determining true values of corticosterone is low for commercial ELISA kits, they have proven to be useful to determine relative differences within studies (e.g. head-fixed animals vs freely moving animals vs sham animals).

## Analysis

*In vivo* Ca^2+^ imaging using GRIN lenses and 1-photon-based microscopes enables the study of neural activities in behaving animals which would be impossible using other techniques. However, the high and fluctuating background fluorescence, inevitable movement and distortion of the imaging field, and extensive spatial overlaps of fluorescent signals emitted from imaged neurons, present major challenges for extracting neuronal signals from the raw imaging data (Lu *et al.* 2018). Overall, the experimenter will need to identify the spatial outline of each neuron in the field of view, untwine spatially-overlapping neurons, and finally deconvolve the spiking activity of each neuron from the dynamics of the Ca^2+^ indicator.

### Enhancement and motion correction

Overall, several steps are necessary to obtain valuable signals. First, the data needs to be ‘enhanced’ by applying a spatial filter that enhances the signal intensity and removes as much background noise as possible. It is then necessary to perform movement correction in order to align (or register) images. This step not only helps correct for movement that can occur when animals are grooming, chewing etc., but also helps with brain tissue movement in areas close to ventricles. Indeed, we have observed that the brain shows movement even when animals are static, which we hypothesise is due, at least in part, to cerebral spinal fluid flowing through the ventricles. Non-rigid motion-correction algorithms that run on Fiji (such as Moco (Dubbs *et al.* 2016)) or Matlab (such as NoRMCorre (Pnevmatikakis & Giovannucci 2017)) have proven useful in solving large-scale image registration problems.

### Extraction of calcium signals in regions of interest

When images have been treated and motion corrected, they are ready to be analysed. Fluorescence in regions of interest (ROI) can be measured over time. The selection of ROI can easily be done by hand (ROI Manager in Fiji) in very scattered populations, but for large data sets that contain a dense populations of neurons, automated cell-sorting algorithms may be better suited to extract spatial and temporal properties of individual cellular Ca^2+^ signals (Mukamel *et al.* 2009, Pnevmatikakis *et al.* 2016). In brief, most cell identification algorithms run principal component analysis (PCA) to remove signals that mostly encode noise. Dimension reduction is then followed by independent component analysis (ICA) to identify the spatial and temporal properties of visualized cells by segmenting the data into statistically independent spatial and temporal signals. PCA/ICA relies on statistical independence of signals and fluorescent signals will be identified as cells only if their activities are not synchronised and if they do not have the same pixel coordinates. The extracted signals can then be manually sorted to confirm that they represent cellular Ca^2+^ transients from spatially defined GCaMP-expressing cells. This technique is particularly helpful for the analysis of dense populations and allows the rapid identification of thousands of neurons (Ziv *et al.* 2013, Jennings *et al.* 2015), but has also been used to successfully extract individual cellular signals from relatively sparse neuronal populations such as AGRP neurons located within the arcuate nucleus (Betley *et al.* 2015). Alternatively, constrained nonnegative matrix factorization (CNMF) approaches have been developed for *in vivo* Ca^2+^ recording analysis (Zhou *et al.* 2018) that have proven to be more accurate than PCA/ICA methods. The main advantage is that CNMF methods have the capability of demixing spatially overlapping neurons. The CNMF-E (Constrained Nonnegative Matrix Factorization for microEndoscopic data) variant has an added model to estimate and account for time-varying background fluctuations in fluorescence (Zhou *et al.* 2018, Aharoni & Hoogland 2019). It is however important to note that all computational methods also have limitations that can reduce the accuracy of data interpretation (Resendez *et al.* 2016), and it is currently unclear which method is best suited for Ca^2+^ indicator data from epifluorescence deep-brain imaging. For a complete review of Ca^2+^ imaging data analysis and alternative techniques see (Pnevmatikakis 2019).

### Spike detection

Spike inference methods aim to estimate the spike times of a neuron given its isolated fluorescence trace. After ROIs of each active neuron have been identified, δF/F traces can be extracted then deconvolved to approximate spiking activity of each segment. This process can be challenging due to the unknown and often non-linear nature of the indicator dynamics, the presence of measurement noise, and the relatively low imaging rate of typical recordings (Pnevmatikakis 2019). While CNMF methods have been successfully used to infer spikes, it should be emphasized that the signal reported by a fluorescent indicator is a convolution of the Ca^2+^ transients and the indicator response. Although a good temporal relationship between neuronal firing and GCaMP signals has been reported for several neuronal cell types (Chen *et al.* 2013, Dana *et al.* 2019), this relationship cannot be taken for granted and Ca^2+^ transients should not be seen as electrical recordings. In order to help with the interpretation of *in vivo* GCaMP fluorescence dynamics in specific neuronal populations, it can be informative to undertake dual electrical-Ca^2+^ imaging experiments in targeted neurons *in vitro* before performing *in vivo* experiments.

### Analysis packages

To facilitate the management of big data sets as well as data analysis by non-experts, open access analysis packages have become available and are constantly improving. The following three packages have been used successfully by the miniscope community: (1) CaImAn, a computational toolbox for large scale calcium imaging analysis, which includes movie handling, motion correction, source extraction, spike deconvolution and result visualisation (Giovannucci *et al.* 2019), which is the most up-to-date package; (2) MiniscoPy, a python-based package adapted from CaImAn specifically to analyse miniscope recordings (Zhou *et al.* 2018); and (3) MiniscopeAnalysis, an updated version of the initial miniscope analysis package developed for the miniscope project. As an alternative to these packages, Lu and colleagues (Lu *et al.* 2018) have developed a fully automated algorithm called the miniscope 1-photon imaging pipeline (MIN1PIPE), which contains several stand-alone modules and can handle a wide range of imaging conditions and qualities with minimal parameter tuning, and automatically and accurately isolate spatially localized neural signals. We have worked with MIN1PIPE and found it efficient and easy to use and adapt to our needs without having to be a specialist in data analysis.

Inscopix clients will benefit from the user-friendly ‘Inscopix Data Processing’ software that performs all steps of the analysis (filter, motion correction, cell registration for longitudinal studies, extraction of the signal) with very limited parameter tuning. We have found it very easy to use and convenient for recordings with good SNR and limited motion artefacts.

### Computer/hard disk specifications

The high-definition data acquired during Ca^2+^ imaging sessions require a significant amount of hard disk space during recording (acquiring 30 min of data at the frame rate of 20 Hz with the nVista miniscope requires approximately 110 GB of hard disk space) as well as a substantial amount of space for data storage. This problem should be thought through before starting any recordings. Moreover, in order to perform longitudinal studies, movies from different recording sessions will need to be concatenated into a single large file, and powerful computers with numerous cores will be required for analysis.

## Readout and physiological consequence of the signals recorded

Once the recordings have been analysed and spatial and temporal data have been extracted, it can still be a challenge to understand the signals. Indeed, within a defined neuronal population, subgroups or individual neurons may be active at different time points, so it is critical to find ways to distinguish the activity we are interested in from the rest.

### Behavioural/environmental changes

**Figure 4:**
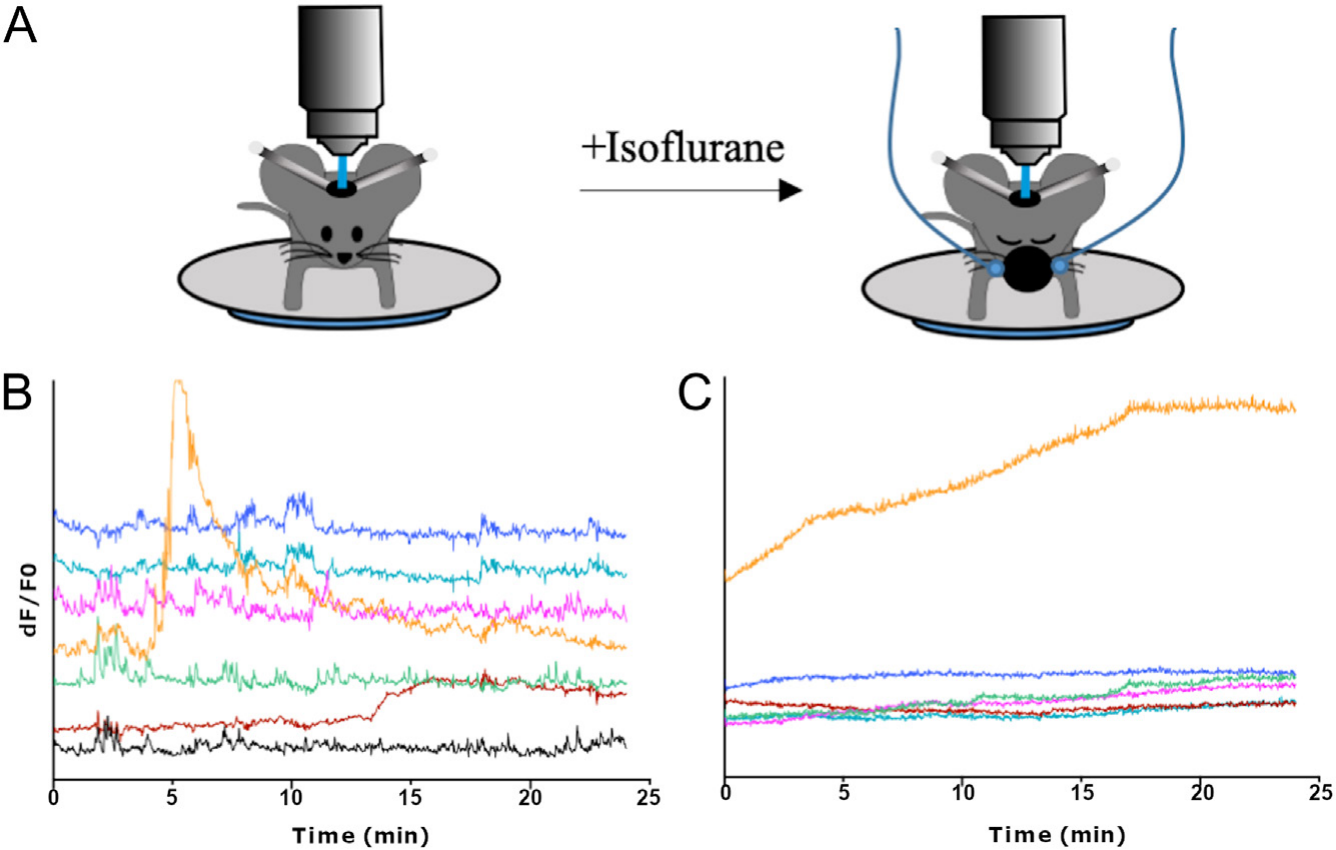
Isoflurane changes the calcium activity of arcuate nucleus neurons (A) 25-min-long *in vivo* recordings of arcuate nucleus neurons were performed in head-fixed mice, mice were then anaesthetised with isoflurane and the same neurons were recorded for another 25 min. (B) Time-lapse recordings of calcium (GCaMP6s) activity of 7 arcuate nucleus neurons in an awake adult C57Bl6 male. (C) Time-lapse recordings of calcium activity of the same 7 arcuate nucleus neurons in the same anaesthetised adult male.

One of the most notable benefits provided by *in vivo* imaging of neuronal activity in conscious animals is that it is possible to correlate the signal with contextual changes in environment (e.g. time of the day, temperature, light intensity), behaviour (e.g. environmental exploration, time-locked stimulus presentations), or pharmacological induced alterations of network activity. Indeed, the ability to image cell type-specific activity during naturalistic animal behaviour allows this imaging protocol to be applied to a wide array of experimental manipulations, not only to answer questions that could not be tackled using previous methods for monitoring of neural activity, but to provide clues about the significance of the recorded calcium signals. With the ability to record activity across several days/weeks, each animal can become its own control. But what is perhaps more valuable is that because individual neurons can be registered and therefore studied over several recording sessions, each neuron can become its own control. We have used this experimental design to study the effect of isoflurane on hypothalamic neuronal activity. General anaesthetics, especially isoflurane, are routinely used to induce a sleep-like state, allowing otherwise painful or stressful procedures to be performed, but have been long known to alter hormonal secretion. For example, in rats fed *adlibitum*, isoflurane increases glycemia, glucagon and GH levels but causes decreases in insulin, ACTH and corticosterone levels (Saha *et al.* 2005). Similarly, pulsatile LH secretion and LH concentrations are diminished in anaesthetised mouse models (Campos & Herbison 2014). How exactly isoflurane works in the brain is not fully understood (Campagna *et al.* 2003), and although its effect on neurons has not been studied in real-time *in vivo*, studies have reported disrupted calcium activity in astrocytes (Thrane *et al.* 2012) and altered blood flow in the brain (Rungta *et al.* 2017) following isoflurane anaesthesia. Armed with the ability to image hypothalamic neurons *in situ*, we have been able to characterise the effect of isoflurane on the Ca^2+^ dynamics of arcuate nucleus neurons. We performed Ca^2+^ imaging in head-restrained mice first in awake conditions, and then for a 25-min period following anaesthesia induction. We monitored the same neurons in both conditions and found that isoflurane greatly altered the Ca^2+^ activity of the recorded neurons (Fig. 4) (P Campos and P Mollard, unpublished observations).

### Blood sampling

An important advantage of working with neuroendocrine circuits is that the final readout of the network and its physiological relevance can in many cases be readily assessed through changes in circulating hormone concentrations. The recent advance in ultra-sensitive hormone assays for pituitary hormones (Steyn *et al.* 2011, 2013, Guillou *et al.* 2015) have allowed researchers to increase the sampling frequency while decreasing blood volumes needed to get a high-resolution reading of hormonal dynamics. Two main techniques can be effectively combined with *in vivo* imaging of hypothalamic neurons.

#### Tail-tip method

The tail-tip technique involves cutting the last 1 mm of tail tip and then collecting small 3–5 μL volumes of blood that can be analysed for hormone concentration using ultra-sensitive assays. The blood flow is encouraged by gently massaging the tail and stops spontaneously when stroking is stopped. Because of the size of the samples this technique results in minimal blood loss and has been shown to be very efficient in characterising LH and GH profiles in mice (Steyn *et al.* 2011, 2013). To succeed in using this blood sampling method thorough training is necessary, particularly as animals will be either tethered or head-restrained. Animals need to get used to staying in the hand of the experimenter, being restrained by holding the base of the tail, and having their tail massaged. It is important to note that rats are more sensitive than mice with their tail, and we have failed to measure LH surges in female rats after taking frequent blood samples using this technique.

#### Automated blood sampling

To collect a series of blood samples at high frequency, animals can be implanted with vascular cannula(s) and connected to an automated blood sampling (ABS) system. These systems withdraw small blood samples via the vascular cannula, which are sent to a fraction collector where they are deposited into collection tubes. The advantage of ABS systems is that the blood sampling procedure can be carried out with minimal disturbance to the animal, and the sampling frequency can be specified according to the goal of the study. These experiments are considered stress-free for the animals and have been used extensively to study the regulation of the hypothalamic-pituitary-adrenal axis (Windle *et al.* 1998, Walker *et al.* 2012). There are, however, some disadvantages to this technique. First, the animals need to be implanted with a vascular cannula placed in the jugular vein. Because of the difficulty of this surgery, these experiments, although possible in mice (Adams *et al.* 2015) are better-suited to larger rodents such as rats. Second, the sample volumes are typically greater than those collected using the tail-tip technique. Third, this technique is particularly suited for the measurement of stable hormones such as corticosterone (Walker *et al.* 2012). Finally, as animals need to be tethered to perform ABS studies, attention is required to ensure the cable from the miniscope does not become tangled.

## Conclusions

Deep-brain imaging techniques have the potential to produce data that were long thought impossible to obtain because of the inaccessibility of the hypothalamic-pituitary system. Overall, being able to study the activity of specific hypothalamic neurons in awake animals and in real time offers an incredible and invaluable opportunity to broaden our understanding of how the brain controls endocrine function. It also gives us the opportunity to study why endocrine functions become disrupted in pathophysiological conditions, and how these changes may lead to the development or even protection from pathological consequences. Furthermore, *in vivo* brain imaging in conscious animals permits longitudinal studies, thus offering the capability to use animals (and even individual neurons) as internal controls prior to a challenge (e.g. stress, pregnancy, circadian perturbation), which is in-line with 3Rs guidelines and compares favourably with approaches involving numerous animals to provide adequate *n* numbers in test and control groups. While these techniques were long thought to be available only to laboratories specialised in optics and engineering, or to laboratories with sufficient funding to purchase ready-made equipment, they are now assessible to the wider endocrine field with the help of open source resources. It is expected that high-quality research and high-impact publications emerging from these methodologies will benefit the scientific and medical communities and open up new research opportunities. While this review has focused on imaging Ca^2+^ dynamics within neurons, many other indicators exist. Of particular interest are Flamindo (Odaka *et al.* 2014) and R-FlincA (Ohta *et al.* 2018), which are single-wavelength indicators that permit imaging of 3′5′-cyclic adenosine monophosphate (cAMP) dynamics in living organisms. Likewise iATPSnFRS allows imaging of extracellular and cytosolic ATP (ATP) in astrocytes and neurons (Lobas *et al.* 2019). Intensive efforts have also resulted in the development of modified G-protein-coupled receptors in which a permuted GFP has been inserted into the third loop in order to monitor various signalling molecules. While this technology currently only works for the detection of small neurotransmitters such as acetylcholine (Jing *et al.* 2018) and dopamine (Patriarchi *et al.* 2018), one can hope that these tools will soon be suitable for *in vivo* detection of peptides and neuropeptides.

## Declaration of interest

The authors declare that there is no conflict of interest that could be perceived as prejudicing the impartiality of this review.

## Funding

P C and J J W acknowledge financial support from the Medical Research Council (fellowship MR/N008936/1 to J J W) and the Engineering and Physical Sciences Research Council (grant EP/N014391/1 to J J W). P C and P M acknowledge financial support from the Agence Nationale de la Recherche (ANR-15-CE14–0012-01, ANR-18-CE14–0017-01), France-Bioimaging (INBS10-GaL/AR-11/12), Institut National de la Santé et de la Recherche Médicale, Centre National de la Recherche Scientifique, Université de Montpellier, and Fondation pour la Recherche Médicale (DEQ20150331732).
